# TGF-β pathways in aging and immunity: lessons from *Caenorhabditis elegans*

**DOI:** 10.3389/fgene.2023.1220068

**Published:** 2023-09-05

**Authors:** Katerina K. Yamamoto, Cathy Savage-Dunn

**Affiliations:** Department of Biology, Queens College, and PhD Program in Biology, The Graduate Center, City University of New York, New York City, NY, United States

**Keywords:** aging, innate immunity, TGF-β, BMP, *C. elegans*

## Abstract

The Transforming Growth Factor-β (TGF-β) superfamily of signaling molecules plays critical roles in development, differentiation, homeostasis, and disease. Due to the conservation of these ligands and their signaling pathways, genetic studies in invertebrate systems including the nematode *Caenorhabditis elegans* have been instrumental in identifying signaling mechanisms. *C. elegans* is also a premier organism for research in longevity and healthy aging. Here we summarize current knowledge on the roles of TGF-β signaling in aging and immunity.

## Introduction

As organisms age, they undergo gradual cellular and molecular changes, accompanied by a decline in many physiological functions. Consequently, their susceptibility to age-related diseases and conditions increases (López-Otín et al., 2013; Son et al., 2019; Melzer et al., 2020). Many fundamental findings in the field of aging have come from studies in the small free-living nematode *C. elegans* (Murphy and Hu, 2013). *C. elegans* has been used as a model organism for decades due to its short lifespan of approximately 3 weeks, small size, transparent body, easy laboratory maintenance, genetic tractability, and conserved biological pathways (Brenner, 1974; C. elegans Sequencing Consortium, 1998). Approximately 83% of the *C. elegans* proteome has human homologs (Lai et al., 2000), and over 50% of human protein-coding genes have homologs in *C. elegans* (Sonnhammer and Durbin, 1997; Kuwabara and O’Neil, 2001; Harris et al., 2004). The insulin/IGF-1-like signaling pathway (IIS) was the first pathway identified to regulate lifespan in *C. elegans*. Mutations in *daf-2*, subsequently found to encode the sole Insulin/IGF-1-like receptor (Kimura et al., 1997), result in a doubled lifespan compared to wildtype (WT) (Kenyon et al., 1993). Further studies in *C. elegans* revealed the roles of other pathways that regulate aging including AMP-activated protein kinase (AMPK), and mechanistic target of rapamycin (mTOR) (Zhang et al., 2020). In addition, transforming growth factor beta (TGF-β) pathways are emerging as regulators of longevity and healthy aging that warrant further study.

One of the systems that faces the most consequential impacts of aging is the immune system, where age-associated decline is called immunosenescence. This decline manifests in increased infection susceptibility, decreased vaccination response, and increased risk for cancer and autoimmune diseases. The underlying changes that result in these physiologies in mammals are: a decrease in immune cell repertoire, cell intrinsic defects to lymphocytes, and increased inflammation (Akha, 2018). Aging and immunity can be regulated by shared molecular mechanisms, such as IIS, TGF-β, mTOR and nuclear factor kappa B (NF-κB) pathways (Powell et al., 2012; Papadopoli et al., 2019; Songkiatisak et al., 2022). Promoting healthy aging will need to address immunosenescence for an improved quality of life.

In addition to its pioneering contributions to the study of aging, *C. elegans* is also a valuable model system to study innate immunity (Nicholas and Hodgkin, 2002; Millet and Ewbank, 2004; Schulenburg et al., 2004). The animals’ diet consists of available bacteria in their environment, which in the laboratory is a non-pathogenic strain of *Escherichia coli*, OP50. This system is easily modified to study immunity, since the food source can be replaced with pathogenic bacteria, which allows bacterial pathogens to be easily introduced to the species. Additionally, *C. elegans* do not have antibody-based acquired immunity that could confound studies on innate immunity. As such, studies with *C. elegans* have identified conserved signaling pathways that regulate innate immunity, including IIS, p38 mitogen-activated protein kinase (MAPK) and TGF-β signaling (Ewbank, 2006; Kim and Ewbank, 2018). Immunity pathways overlap significantly with those that regulate aging (Kurz and Tan, 2004; Fabian et al., 2021). For example, mutations in DAF-2 and other IIS components not only extend lifespan, but also have improved resistance against bacterial pathogens than WT animals, although these effects can be uncoupled (Garsin et al., 2003; Kerry et al., 2006; Evans et al., 2008; Lee et al., 2021). Future studies are needed to expound these inter-connected physiologies, where improved understanding will positively contribute to healthy aging.

## TGF-β signaling

The TGF-β superfamily of extracellular signaling molecules is an ancient and conserved mechanism of cell-cell communication in animals (Robertis, 2008). Disruptions in TGF-β signaling result in birth defects, as well as autoimmune disorders, cancer, and other diseases (Padua and Massagué, 2009; Wu and Hill, 2009). There are two major groups within the TGF-β superfamily, TGF-β/Activin and bone morphogenetic proteins (BMPs) (Herpin et al., 2004). TGF-β has known roles in cell proliferation, differentiation, apoptosis and reproductive function. For example, anti-müllerian hormone is required for follicular development in females, and when dysregulated, can cause polycystic ovarian syndrome, development of female reproductive structures in males, among others (Wu and Hill, 2009). Activin also plays a role in cell proliferation, differentiation, apoptosis, as well as reproductive function, commonly known for regulating many parts of the menstrual ovulatory cycle in humans (Adu-Gyamfi et al., 2020). Nodal is another member of the TGF-β/Activin group that is involved in embryonic development, such as axis formation and patterning (Wu and Hill, 2009). BMPs were first identified for their role in regulating bone and cartilage development (Urist and Strates, 1971; Wang et al., 1988; Wozney et al., 1988; Herpin et al., 2004). They are best known for roles in development and differentiation, such as embryonic body plan patterning and cell identity specification, but are emerging as modulators of homeostasis.

There is strong evidence that TGF-β signaling regulates aging, particularly in several age-associated diseases. TGF-β2 and the TGF-β receptors (I and II) have decreased expression in the cartilage of knee joints of old mice, compared to young mice. Blocking TGF-β activity demonstrated that TGF-β is necessary for a normal repair response, thus causing cartilage tissue damage (Davidson et al., 2005). This role has substantial implications in osteoarthritis, a prevalent age-associated disease. Another instance of age-related expression changes is BMP4, which was shown to increase with age in the dentate gyrus of both mice and humans (Meyers et al., 2016). The dentate gyrus is a part of the hippocampus that functions in learning and memory, and typically declines during aging. To establish a better relationship between cognitive function and BMP4 levels, a BMP4-expressing lentivirus was injected, which resulted in decreased cognition, compared to mice who had a control virus injected (Meyers et al., 2016). This function likely has implications in age-associated diseases that impair cognition, such as Alzheimer’s disease, and warrants continued study.

An enticing 2013 study found that TGF-β family member GDF11 could act as a circulating factor that reverses age-related decline (Loffredo et al., 2013). A conflicting report found that GDF11 levels increase rather than decrease during aging, and that the previous conclusions could have been confounded by reagents that cross react between GDF11 and GDF8 (myostatin) (Ahlberg et al., 2015). Controversy continues to the present day regarding the action of GDF11 in aging, a testament to the complexity of TGF-β signaling in the mammalian context (Ma et al., 2021). A less controversial GDF ligand is GDF-15, which has established roles in both immunity and aging (Pence, 2022). GDF-15 is a stress-induced cytokine, which acts as an immunoregulatory protein, dampening inflammatory responses in many immune cell types under pro-inflammatory conditions. Interestingly, GDF-15 has been shown to increase with age. One study examined the plasma proteome over lifespan and found that GDF-15 was the single protein most associated with age, increasing linearly.

TGF-β members also regulate immunity. Of the three TGF-β members in humans, TGF-β1 is most involved in immunity. TGF-β1 is a major regulator of T-cell development, homeostasis, and survival (Li and Flavell, 2008). In fact, mice lacking functional TGF-β1 are severely immunodeficient (Gough et al., 2021), and eventually develop fatal inflammatory diseases, even under germ-free conditions (Li and Flavell, 2008). Another TGF-β family ligand is Activin A, from the Activin subgroup, which plays a role in both innate and adaptive immunity. Based on cell type and context, Activin A can function as anti-inflammatory or pro-inflammatory, and is very widespread in its immune regulation (Chen and Dijke, 2016). The aging and immunity functions of TGF-β signaling intersect with TGF-β′s well-known role in cancer regulation. During homeostasis, TGF-β signaling maintains a healthy cellular environment. However, during tumor progression, this signaling can become disrupted, instead suppressing immune systems, promoting cancer (Batlle and Massagué, 2019). This relationship between cancer and immunity is largely through adaptive immunity, which *C. elegans* does not have, however there are some aspects of innate immunity involved, such as the inhibition of natural killer cells, and regulating macrophages.

The canonical signal transduction pathway begins with a TGF-β ligand dimer binding to a heterotetrameric complex composed of type I receptor and type II receptors (Dijke and Hill, 2004). The type II receptors’ serine/threonine kinase domain phosphorylates the type I receptors’ glycine-serine (GS) domain, which activates the type I receptor serine/threonine kinase. This kinase activity then phosphorylates receptor regulated Smads (R-Smads) at the C-terminus, which form a heterotrimeric complex with common mediator Smads (Co-Smads). The Smad complex enters the nucleus to regulate gene transcription (Massagué et al., 2005). TGF-β/Activin and BMP ligands signal predominantly through different receptors and Smads: Smad2/Smad3 act with Smad4 for TGF-β/Activin and Smad1/Smad5/Smad8 act with Smad4 for BMPs (Dijke and Hill, 2004).

## TGF-β signaling in *Caenorhabditis elegans*-Overview

**FIGURE 1:**
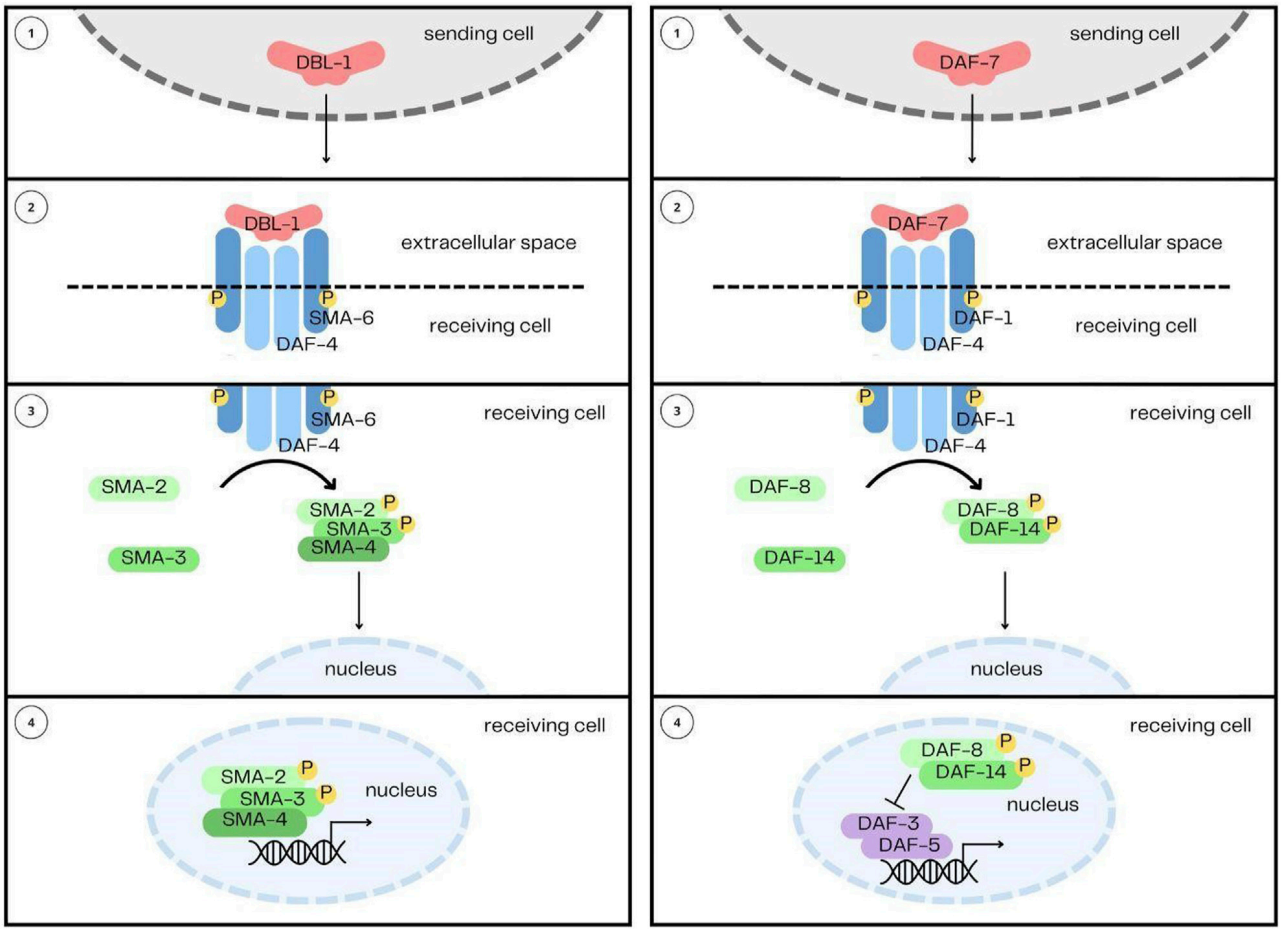
The DBL-1/BMP and DAF-7/TGF-β Signaling Pathways. The two pathways share their overall structure in common: 1) the ligands, DBL-1 and DAF-7, are secreted from their sending cells, 2) and are received by heterotetrameric complexes comprised of two type I receptors and two type II receptors. 3) Signal is then transduced by Smads, 4) which enter the nucleus and interact with transcription factors to regulate gene expression.

In the *C. elegans* genome, there are five genes encoding TGF-β family ligands: DBL-1, DAF-7, TIG-2, TIG-3 and UNC-129 (Gumienny and Savage-Dunn, 2013). In comparison, humans have over 30 TGF-β family members. The reduced complexity of TGF-β signaling in *C. elegans* is an experimental advantage for elucidating the functions and signaling mechanisms of the pathways (Table 1). DBL-1 and TIG-2 are members of the BMP family; DAF-7 and TIG-3 are related to TGF-β/Activin; and UNC-129 is more divergent (Savage-Dunn and Padgett, 2017). Two of the ligands, DBL-1 and DAF-7, have well-defined signaling pathways as described further below (Figure 1). While DAF-7 signaling has been associated with longevity since 2007 (Shaw et al., 2007), recent advances have demonstrated roles for all of these ligands in immunity (Ciccarelli et al., 2023b).

**TABLE 1:** TGF-β ligands in *Caenorhabditis elegans*.

Pathway component	*C. elegans* component	Putative human homologs	Aging	Refs.	Microbiome	Refs.	Immune response	Refs.
Ligand	DBL-1	BMP2, BMP4, BMP5, BMP6, BMP8, BMP10, GDF3, GDF5, GDF7	Plays a minor role regulating somatic aging, moderate role regulating lifespan in sensitized backgrounds, however a major role in reproductive aging.	Mallo et al. (2002), Luo et al. (2009), Luo et al. (2010), So et al. (2011)	Mediates lifespan extension or increased pathogen resistance by some gut microbes. Also influences microbiome selection.	Kwon et al. (2016), Kwon et al. (2018), Berg et al. (2019), Haçariz et al. (2021), Mørch et al. (2021), Zhang et al. (2021a), Zhou et al. (2021), He et al. (2023)	Major regulator of innate immunity by modulating behavior, barrier functions and transcriptional regulation of AMPs in response to pathogens. Experiences increased susceptibility to infection.	Mallo et al. (2002), So et al. (2011), Mochii et al. (1999), Alper et al. (2007), Liang et al. (2007), Tenor and Aballay (2008), Zugasti and Ewbank (2009), Roberts et al. (2010), Portal-Celhay et al. (2012), Zhang and Zhang (2012), Julien-Gau et al. (2014), Madhu et al. (2020), Madhu and Gumienny (2021), Ciccarelli et al. (2023a)
	DAF-7	Activin, Inhibin, GDF8, GDF11, GDF15	Plays a significant role in somatic aging, however minimal evidence that it is involved in reproductive aging.	Shaw et al. (2007)	-	-	Major regulator of innate immunity largely by modulating pathogen avoidance behavior with limited involvement in transcriptional response.	Meisel et al. (2014), Harris et al. (2019), Moore et al. (2019), Singh and Aballay (2019a), Singh and Aballay (2019b), De-Souza et al. (2022)
	TIG-2	BMP2, BMP4, BMP5, BMP6, BMP8, BMP10, GDF3, GDF5, GDF7	-	-	-	-	Regulator of immune response.	Ciccarelli et al. (2023b)
	TIG-3	Activin, Inhibin, GDF8, GDF11, GDF15	-	-	-	-	Regulator of immune response.	Ciccarelli et al. (2023b)
	UNC-129	-	-	-	-	-	Regulator of immune response.	Ciccarelli et al. (2023b)

## The DBL-1/BMP pathway

The DBL-1 pathway was first identified from two mutant phenotypes: small body size (Sma) and male abnormal tail morphology (Mab) (Baird and Ellazar, 1999; Morita et al., 1999; Suzuki et al., 1999; Savage-Dunn et al., 2003), hence also giving the alternative pathway name Sma/Mab (Savage et al., 1996; Padgett et al., 1998). The DBL-1 pathway is not essential for organism viability, which has made many genetic manipulations and further studies possible. The DBL-1 signaling pathway, since its original identification, has been demonstrated to play a role in innate immunity, reproductive aging, mesodermal patterning, chemosensation, L1 development, and lipid metabolism (Mallo et al., 2002; Foehr et al., 2006; Vashlishan et al., 2008; Almedom et al., 2009; Luo et al., 2009; Kaplan et al., 2015; Yu et al., 2017; Clark et al., 2018). This pathway in *C. elegans* follows the canonical signal transduction pathway described earlier and begins with the ligand DBL-1, named for being Dpp and BMP-like (Morita et al., 1999; Suzuki et al., 1999). The signal from DBL-1 is received by a heterotetramer formed by the type I receptor SMA-6 (Krishna et al., 1999), and the type II receptor DAF-4 (Estevez et al., 1993). These two receptors have different recycling mechanisms (Gleason et al., 2014), regulated by tetraspanins (Liu Z. et al., 2020). Signal is then transduced by the receptor-regulated Smads, SMA-2, SMA-3, and the common mediator Smad, SMA-4 (Savage et al., 1996). Null mutants of *sma-3* display phenotypes equally as severe as *dbl-1* null alleles (Savage-Dunn et al., 2000), indicating a lack of redundancy between the Smads in the pathway. Downstream transcription factors include SMA-9, the homolog of *Drosophila* Schnurri (Liang et al., 2003), LIN-31/forkhead (Baird and Ellazar, 1999) and MAB-31 (Wong et al., 2010). DBL-1 is expressed and released by cholinergic neurons in the head and nerve cords (Morita et al., 1999; Suzuki et al., 1999; Duerr et al., 2008). This includes the AFD amphid neuron, ventral nerve cord, DA, DB, VA, and VB motorneurons, several neurons in the pharynx, and in the male tail, the spicule socket cells and the DVA neuron (Morita et al., 1999; Suzuki et al., 1999). An upstream transcription factor regulating *dbl-1* expression is UNC-3 (Kratsios et al., 2012). DBL-1 receptors and Smads are expressed in the epidermis (hypodermis), pharynx and intestine (Patterson et al., 1997; Krishna et al., 1999; Yoshida et al., 2001; Mallo et al., 2002; Wang et al., 2002; Reece-Hoyes et al., 2007).

## DBL-1 and aging

The DBL-1 pathway plays a major role in reproductive aging (Luo et al., 2009; Luo et al., 2010). In WT hermaphrodites, the mean reproductive span is 3.5 days, while in *dbl-1* mutants, it is greater than 7 days (Luo et al., 2009). Other BMP pathway mutants, such as *daf-4*, *sma-2*, *sma-3* and *sma-9*, also have increased reproductive spans (Luo et al., 2009), suggesting that the DBL-1 pathway is responsible for correctly timing cessation of reproduction. The regulation of reproductive span is uncoupled from longevity, and independent of DAF-2/Insulin signaling and dietary restriction because the phenotypes in DBL-1 pathway mutants do not require DAF-16 or PHA-4 (Luo et al., 2009). Inactivation of DBL-1 signaling delays cessation of reproduction by maintaining oocyte and germline quality non-cell autonomously. In *sma-2* mutants, proliferating germ cells are maintained for longer. In aged adults, these mutants have improved oocyte morphology compared to WT, which results in improved embryo quality, and less embryonic lethality than WT. *sma-2* mutant adults also have improved distal germline integrity, with fewer indicators of decline, such as cavities, graininess and cellularization (Luo et al., 2010). A genome-wide RNAi screen identified 32 genes that, when inactivated, extend reproductive span (Wang et al., 2014). 25 of these 32 genes were shown to interact with SMA-2 to enact this phenotype (Wang et al., 2014). Later work elucidated that inactivation of DBL-1 signaling reduces cAMP response element-binding protein (CREB) activity downstream. This reduction elevates the Hedgehog-related WRT-10, which interacts with Patched-related receptors PTC-1 and PTR-2 to promote oocyte quality maintenance during aging and prevent “normal” cessation of reproduction (Templeman et al., 2020). All core components of DBL-1/BMP signaling have been shown to regulate the *vit-2* vitellogenin enhancer (Goszczynski et al., 2016). As a result, loss-of-function BMP mutations could have decreased vitellogenin transcription causing slower oocyte yolk accumulation and oocyte maturation time, however further experimental studies are needed. This could be a partial explanation for the extended reproductive span of these mutations.

While DBL-1 signaling plays a major role in reproductive aging, in somatic aging, the pathway plays a minor to moderate role, depending on genetic background. In different experiments, *dbl-1* mutant animals have slightly reduced or slightly increased lifespan compared to WT (Mallo et al., 2002; Luo et al., 2009; So et al., 2011). Reduced lifespan may be due to an immune defect, where *dbl-1* mutants are more sensitive to the mild pathogenic effects of OP50 (McCulloch and Gems, 2003; Luo et al., 2009; Haque and Nazir, 2016). Consistent with that hypothesis, *dbl-1* mutants have an increase in survival on heat-killed OP50 *versus* live OP50, however not enough to return lifespan to WT levels on heat-killed OP50 (Mallo et al., 2002). Furthermore, with the addition of Floxuridine (FUdR), a DNA synthesis inhibitor used to prevent progeny development, the *dbl-1* mutant lifespan was similar to or greater than WT lifespan (Mallo et al., 2002; Luo et al., 2009). The FUdR not only prevents progeny development, but also prevents bacterial replication, likely decreasing the pathogenic effects of OP50. Furthermore, on HB101, a less-pathogenic bacterial strain of *E. coli*, *dbl-1* mutant animals have a comparable lifespan to N2 (So et al., 2011).

In sensitized backgrounds, DBL-1/BMP signaling plays a moderate role in the regulation of longevity. DBL-1 and other BMP signaling components are required for other mutants’ lifespan phenotypes. For example, *daf-2 sma-3* double mutants have a reduced median lifespan when compared to *daf-2* single mutants, indicating that BMP signaling is required to execute this long-lived phenotype (Clark et al., 2021). Another instance of DBL-1 signaling regulating longevity in sensitized backgrounds is with neuronal overexpression of heat shock factor HSF-1, which increases lifespan and reduces BMP pathway activity (Chauve et al., 2021). The lifespan extension requires DBL-1 and SMA-6 because HSF-1 transcriptionally represses DBL-1 by directly binding to the DBL-1 promoter (Arneaud et al., 2022). A third example is that *sma-2* or *sma-3* mutations are required for the longevity of *ifg-1* (initiation factor 4G) mutants (Chomyshen et al., 2022). *ifg-1* mutants have inhibited stress-induced alternative splicing, and upregulated RNA splicing regulators, which extend lifespan. The upregulation of RNA splicing regulators is mediated by SMA-2 (Chomyshen et al., 2022). These three examples demonstrate that BMP signaling components are required to enact the lifespan phenotypes of other mutants by regulating aging genes. Interestingly, SMA-4 was shown to be differentially regulated during aging, which may be an explanation for this regulation (He et al., 2014).

## DBL-1 and the microbiome

The microbiome of *C. elegans* is a topic that intersects aging and immunity, as some microbe species can be beneficial to the host, while other species are detrimental and pathogenic. The DBL-1/BMP pathway has been shown to regulate both beneficial and pathogenic interactions with the microbiome.

Several bacterial strains are known to extend lifespan of *C. elegans*. The human gut microbes *Propionibacterium freudenreichii*, *Butyricicoccus pullicaecorum* and *Megasphaera elsdenii* extend lifespan in WT *C. elegans* (Kwon et al., 2016; 2018). However, *dbl-1* mutant animals had no change in lifespan, indicating that this extension is acting through the DBl-1/BMP pathway (Kwon et al., 2016; 2018). A diet consisting of the bacterial species *Chryseobacterium* sp. CHNTR56 MYb120, from the *C. elegans* native microbiome, also results in lifespan extension, and causes an upregulation in several key DBL-1 pathway components compared to OP50 (Haçariz et al., 2021). Another instance of microbial benefit is from *Lactobacillus* spp. Lb21, which not only extends lifespan when fed to *C. elegans*, but also results in increased resistance against bacterial pathogen methicillin-resistant *Staphylococcus aureus* (MRSA) (Mørch et al., 2021). This increased resistance was dependent on DBL-1 (Mørch et al., 2021). Furthermore, ingestion of certain *Lactobacillus* isolates upregulates the expression of DBL-1 and also p38 MAPK signaling, resulting in increased resistance to *Salmonella typhimurium* dT104 (Zhou et al., 2021).

DBL-1 signaling not only carries out the benefits of some bacterial species, but also influences microbiome selection. In an experiment, animals at the first larval stage were placed on plates with synthetic microbiota, comprising 30 previously isolated *C. elegans* gut commensals in equal parts. After 3 days, *dbl-1* mutants had a threefold increase in gut bacterial load compared to WT, and had an increased abundance of *Enterobacter* species (Berg et al., 2019). When a second synthetic community was tested, *dbl-1* mutants continued to show an expanded gut microbiome, and increased *Enterobacteriaceae*, suggesting these are general qualities of *dbl-1* mutants. *Enterobacter* is typically a commensal bacterial species, however in the context of these compromised mutants, *Enterobacter* becomes pathogenic (Berg et al., 2019). Fascinatingly, WT worms exposed to synthetic microbiota that represent a wild environment experience a bloom in gut *Enterobacteriaceae* during aging (Choi et al., 2023). This bloom results in increased infection susceptibility to *E. faecalis* in the aging population. A causative factor for this *Enterobacteriaceae* bloom turned out to be an age-dependent decline in DBL-1/BMP signaling (Choi et al., 2023). This study highlights how DBL-1/BMP regulation of the microbiome has consequences in aging and immunity. These findings are also supported by mammalian studies. One found that the intestinal microbiomes of patients with inflammatory bowel disease had increased *Enterobacteriaceae* (Morgan et al., 2012). In mice, increased colonic *Enterobacteriaceae* was associated with a loss of TGF-β signaling, suggesting that this role may be conserved (Ihara et al., 2016). In addition to this work, DBL-1 was found to positively regulate the host N-glycosylation protein BCF-1, shaping microbiome selection. BCF-1 directly binds *E. coli* cells using its fimbrial protein, encouraging colonization of the gut by *E. coli* cells (He et al., 2023).

## DBL-1 and immunity

The DBL-1/BMP signaling pathway in *C. elegans* is a major regulator of innate immunity. Early evidence of this role emerged from observations showing an increased susceptibility of *dbl-1* mutants to infection by *Serratia marcescens* (Mallo et al., 2002). In addition, the *dbl-1* pathway component SMA-3 was identified in a genetic screen as having an increased susceptibility to *Pseudomonas aeruginosa* strain PA14 (Tan, 2001). Loss of DBL-1 signaling has consequences in the immune response to a range of pathogens, including bacteria *E. coli*, *Enterococcus faecalis*, *P. aeruginosa* PA14, *Salmonella enterica*, *S. typhimurium* strain SL1344, *S. marcescens*, *Photorhabdus luminescens*, and the nematophagous fungus *D. coniospora* (Mallo et al., 2002; Tenor and Aballay, 2008; Zugasti and Ewbank, 2009; So et al., 2011; Portal-Celhay et al., 2012; Ciccarelli et al., 2023a). It was recently shown that expression of SMA-3 in the pharynx increased survival on pathogenic bacteria compared to *sma-3* mutant animals (Ciccarelli et al., 2023a). In the response to pathogenic fungus *Drechmeria coniospora*, SMA-3 but not SMA-2 or SMA-4 is required, indicating the existence of non-canonical signaling of R-Smad without a Co-Smad (Zugasti and Ewbank, 2009). In trying to identify patterns in how DBL-1 signaling responds to Gram-negative *versus* Gram-positive bacteria, one study tested a panel of three Gram-negative bacteria, and three Gram-positive bacteria, and found trends in DBL-1 pathway activity (Madhu and Gumienny, 2021). Using an integrated fluorescent DBL-1 reporter, they found an induction of DBL-1 signaling on Gram-negative bacteria, while no fluorescence was seen on Gram-positive bacteria. They also identified patterns in avoidance response discussed below. While these trends are interesting, other studies have reported data that contradict this pattern, thus a larger panel of bacteria needs to be studied to understand what DBL-1 signaling patterns exist in response to Gram-negative bacteria or Gram-positive bacteria. SMA-10, an extracellular regulator of the DBL-1/BMP pathway has been shown to regulate immunity, and *sma-10* mutants have increased susceptibility to PA14 infection. Interestingly, SMA-10 seems to be acting independently of DBL-1, instead acting through DAF-2, further demonstrating the extensive crosstalk that exists with IIS (Lucas et al., 2021). All these data suggest that BMP signaling is a broad regulator of immune response to a range of pathogens, however activates different downstream immune effectors in a pathogen-specific manner.

DBL-1/BMP signaling not only regulates immune response, but also is responsible for enacting some consequences of infection. DBL-1/BMP and IIS pathways are responsible for a hormetic effect after developmental exposure to a pathogenic *E. coli* strain (Leroy et al., 2012). Hormesis is a phenomenon where low dose exposure to a typically harmful agent has a beneficial effect on an organism. In a study assessing the impact of bacterial infection on male morphology and spermatogenesis, a 24-h infection with *S. aureus* or *Vibrio alginolyticus* caused abnormal tail morphology and decreased sperm activation (Sharika et al., 2018). The compound pentagalloyl glucose (PGG) is a polyphenol derived from plants. The addition of PGG to *C. elegans* resulted in increased expression of DBL-1 and DAF-4, as well as increased survival on two *P. aeruginosa* strains (Zhang X. et al., 2021). The survival effect was dependent on DAF-4 (Zhang X. et al., 2021).

We can define several mechanisms by which organisms can defend against pathogen exposure. They include: behavior, immune resistance (including innate immunity, which comprises transcriptional regulation of antimicrobial peptides (AMPs) and barrier functions), and tolerance. These mechanisms decrease infection susceptibility and increase survival chance, and operate together as part of a holistic immune response. All of these mechanisms are regulated by the DBL-1/BMP pathway.

The first immune strategy is behavior, which often begins before contact with potential pathogens is made. The primary behavior that supports immunity is avoidance, where a host organism identifies a risk of pathogen exposure, and modulates behavior to decrease this risk. This behavior is well characterized in *C. elegans*, where individuals detect a pathogen, and then distance themselves. A short exposure to certain pathogenic bacteria, such as PA14 and *S. marcescens*, will induce olfactory learning in *C. elegans*, where animals will avoid that bacteria in subsequent exposures (Zhang et al., 2005). This avoidance behavior was shown to require DBL-1 secreted from the AVA command interneurons, and SMA-6 in the epidermis (Zhang and Zhang, 2012). It was later found that production of DBL-1 in ASI and ASJ sensory neurons is prevented by AMPylase FIC-1 overexpression, consistent with their decreased pathogen avoidance (Hernandez-Lima et al., 2022). Loss of *dbl-1* has also been seen to have an increased avoidance of *E. coli*, supporting how OP50 may have increased pathogenicity in these animals compared to WT (Madhu and Gumienny, 2021). In a panel of three Gram-positive bacteria and three Gram-negative bacteria, *dbl-1* mutants displayed strong avoidance to all three Gram-negative bacteria, and a mild response to one of three Gram-positive bacteria. Unexpectedly, loss of SMA-4 had the opposite effect: a severe avoidance response to all three Gram-positive bacteria, correlated with increased SMA-4 expression. This led the authors to hypothesize that while the canonical pathway was involved in responding to all three Gram-negative bacteria, SMA-4 may act independently of DBL-1 in response to some Gram-positive bacteria, perhaps functioning with another pathway (Madhu and Gumienny, 2021). Further studies using a larger bacterial panel would be interesting, and instrumental to make stronger conclusions. *C. elegans* also detect pathogenic bacteria through bacterial secreted peptides, such as serrawettin W2 from *S. marcescens* (Pradel et al., 2007). Given the role of DBL-1 signaling in chemosensation, a fruitful area of future study may be identifying what pathogenic secreted peptides are detected through DBL-1 signaling.

The second immune strategy is the transcriptional regulation of genes involved in the immune response and the induction of AMPs. DBL-1/BMP signaling regulates gene expression of lectins, digestive enzymes like lysozymes and lipases, the PGP (P-glycoprotein) subclass of ATP-binding cassette (ABC) transporter family, caenacin (cnc) AMPs, saposin-like proteins or caenopore AMPs, and the glycocalyx (Mochii et al., 1999; Mallo et al., 2002; Alper et al., 2007; Liang et al., 2007; Zugasti and Ewbank, 2009; Roberts et al., 2010; Julien-Gau et al., 2014; Madhu et al., 2020). One group of AMPs, caenacins, are produced upon infection with a pathogen, and are critical to immune response. DBL-1 was shown to promote *cnc-2* expression in the epidermis, in a dose-dependent manner (Zugasti and Ewbank, 2009). Recent work found that CNC-2, as well as another AMP, ABF-2, are regulated by SMA-3 activity in the pharynx (Ciccarelli et al., 2023a). Another AMP, the saposin-like protein SPP-9, was shown to be negatively regulated by DBL-1 signaling, and is used as a reporter for DBL-1 activity (Roberts et al., 2010; Madhu et al., 2020). The glycocalyx, a glycoprotein and glycolipid exterior layer of many cell membranes, is hypothesized to be involved in immune response. In *C. elegans*, BCF-1 is a believed component of the glycocalyx and is activated upon infection by *P. aeruginosa* and *P. luminescens* (Julien-Gau et al., 2014). BCF-1 requires DBL-1, SMA-6 and PMK-1, demonstrating its regulation by the DBL-1/BMP and MAPK pathways (Julien-Gau et al., 2014). AMPs are induced not only in response to infection, but also by wounding. Wounding was found to activate NAS-38, which enacted the AMP immune response in parallel through the DBL-1/BMP and p38 MAPK pathways (Sinner et al., 2021). Activation of these AMPs increases RIS neuron activity, which promotes sleep and ultimately contributes to survival after pathogen exposure (Sinner et al., 2021). Thus, DBL-1/BMP regulation of AMPs not only directly reduces pathogen load, but also promotes host well-being resulting in improved survival.

The third immune strategy is barrier functions that limit the amount of bacteria that can enter the organism. In some BMP mutants, particularly *dbl-1*, there is more live *E. coli* OP50 present in the gut (So et al., 2011). While this is partially due to the blunted induction of AMPs in *dbl-1* mutants, which enables bacterial colonies to form in the intestine, it may also result from a pharyngeal defect, which allows more live bacteria to enter the intestine. While *dbl-1* mutants appear to have normal pharyngeal pumping and peristalsis, they exhibit abnormal pharyngeal g1 gland cell morphology (Ramakrishnan et al., 2014). Gland cell abnormalities are likely a consequence of DBL-1’s activation of M4, a neuron in the pharynx responsible for initiating peristaltic contractions. The M4 neuron secretes DBL-1, regulated by the upstream homeodomain transcription factor CEH-28. Surprisingly, *sma-2* and *sma-3* mutants did not exhibit any gland cell abnormalities, suggesting that the regulation of gland cell morphology by DBL-1 may be independent of the R-Smads, perhaps instead functioning through a non-canonical pathway (Ramakrishnan et al., 2014). This study was conducted on OP50, so it is not known whether these effects change on more pathogenic bacteria. Later work demonstrated that loss of DBL-1 results in decreased pharyngeal pumping on select pathogens: *Klebsiella oxytoca*, *S. marcescens*, *E. faecalis*, or *S. epidermidis* (Madhu and Gumienny, 2021), suggesting that DBL-1 may be required for the pharynx to function correctly on some pathogens. *sma-3* mutants have been shown to have decreased pharyngeal pumping on *P. luminescens*, which likely contributes to their increased infection susceptibility (Ciccarelli et al., 2023a). This decreased pumping was rescued when *sma-3* was expressed in pharyngeal muscle, demonstrating another instance of DBL-1/BMP regulation of pharynx morphology.

Another physical barrier that is a part of innate immunity is the cuticle, a specialized extracellular matrix that comprises the outermost layer of the animal that separates it from its environment. Specific cuticle collagens that form furrows in the cuticle are responsible for its barrier function (Sandhu et al., 2021). The presence of these collagens enhances the longevity of *daf-2/InsR* mutants (Sandhu et al., 2021); however, their roles in immune barrier function have not yet been tested. DBL-1 is necessary to form the cuticle correctly, and alterations in DBL-1 signaling result in dose-dependent disruptions to cuticle organization and surface lipid content (Schultz et al., 2014). DBL-1 signaling regulates expression of cuticle collagen and extracellular matrix-associated genes (Liang et al., 2007; Luo et al., 2010; Roberts et al., 2010; Yin et al., 2015; Lakdawala et al., 2019), and cuticle collagens regulate DBL-1 signaling (Madaan et al., 2018; 2020), forming a feedback loop that requires further investigation (Goodman and Savage-Dunn, 2022). In *dbl-1* mutants, the permeability of the cuticle is increased (Schultz et al., 2014), although what consequences this increased permeability has on immune response has yet to be assessed.

A fourth defense mechanism is immune tolerance, which aims to reduce the negative consequences that arise from infection. It mitigates detrimental effects caused either directly by the pathogen, or indirectly from host immunopathology resulting from the infection. This mechanism has been better studied in other organisms and needs to be addressed in *C. elegans*. The word “tolerance” itself was chosen for its definition of “the capacity to endure”, demonstrating how this understudied immune strategy may improve survival. This concept was first introduced in plant biology, where organisms from different wheat and oat species had varied tolerance to rust fungi (Caldwell et al., 1958; Schafer, 1971). This concept later was expanded to animal immunity (Ayres and Schneider, 2008; Read et al., 2008) after a study using mice and rodent malaria demonstrated that genetic variation exists for both disease resistance and disease tolerance between individuals, and are independent of one another (Raberg et al., 2009). More recent work from plants and *Drosophila* suggest that lipid metabolism and other homeostatic processes may affect immune tolerance (Lu et al., 2020; Laureano et al., 2021; Deshpande et al., 2022). In *C. elegans*, the fatty acid oleate is required for immune gene transcription, but is not sufficient for pathogen resistance on *P. aeruginosa* PA14, *E. faecalis* and *S. marcescens* (Anderson et al., 2019). The role of oleate, as well as other aspects of lipid metabolism, in immune tolerance is an interesting area that should be further explored. Together, the four mechanisms described have different aims and function at different times: behavior and barrier functions which aim to prevent pathogen load, the induction of AMPs aim to decrease pathogen load, and immune tolerance aims to decrease the burden that infection has on the host once established (Raberg et al., 2007; Medzhitov et al., 2012).

## DBL-1 and response to xenobiotics

Studies on innate immunity have focused on the response to biological pathogens, such as bacteria and fungi. However, the response to non-biological toxins, xenobiotics, overlaps with the immune response. One such xenobiotic is nanoplastics, small pieces of plastic ranging from 1 to 100 nm, which are foreign and potentially harmful substances. It was found that DBL-1/BMP regulates response after exposure (Liu H. et al., 2020). Later work demonstrated that DBL-1 acts downstream of the glutamate receptor GLR-8 (Wang et al., 2021) and the Gα proteins GOA-1, GSA-1 and GPA-10 (Yang et al., 2021).

## The DAF-7/dauer pathway

The DAF-7 pathway was first identified from its regulation of the dauer/continuous development switch, hence giving the alternative pathway name, the Dauer pathway (Riddle et al., 1981; Golden and Riddle, 1984; Thomas et al., 1993). Dauer is a developmental stage that occurs during adverse environmental conditions, such as high population density, or low food availability. Entering this stage results in morphological changes to the organism, including an elongated body, narrowed pharynx, as well as metabolic changes, which allow the animals to survive for several months without food and water. The duration they can survive in the dauer state far exceeds the typical lifespan of *C. elegans*, which is approximately 3 weeks. DAF-7 interacts with DAF-2/insulin signaling to regulate dauer entry and exit. In addition to its role in dauer formation, the DAF-7 pathway also functions in lipid metabolism, feeding behavior, aging, the germline proliferative zone, and autophagy (Fujiwara et al., 2002; Hirose et al., 2003; Greer et al., 2008; Park et al., 2010; Dalfó et al., 2012; Lee et al., 2017; Zhang et al., 2019). This pathway in *C. elegans* begins with the ligand DAF-7 (Ren et al., 1996). The ligand is received by a heterotetramer formed by the type I receptor DAF-1 (Georgi et al., 1990) and the type II receptor DAF-4 (Estevez et al., 1993). Signal is then transduced by the receptor-regulated Smads DAF-8 (Park et al., 2010) and DAF-14 (Inoue and Thomas, 2000), and common mediator Smad, DAF-3 (Patterson et al., 1997). DAF-5, the Sno/Ski homolog, acts downstream of the DAF-7 pathway and binds DAF-3 in a conserved manner (Graca et al., 2003; Tewari et al., 2004; Inoue and Imamura, 2008). Components of the DAF-7 pathway have diverged from TGF-β components in other organisms, but retain sequence features in common and may be more related to TGF-β/Activin components than to BMP components.

DAF-7 expression is detected primarily in ASI sensory neurons (Ren et al., 1996; Schackwitz et al., 1996), however other pathway components are more widely expressed. DAF-7 expression in ASI sensory neurons is repressed in unfavorable conditions, such as high population density and food scarcity. This repression contributes to the formation of dauer in these conditions (Ren et al., 1996). Consequently, null mutant alleles of DAF-7 display a dauer constitutive phenotype.

## DAF-7 and aging

DAF-7 plays a significant role in somatic aging. Early reports concluded that DAF-7 and IIS pathways diverge in adulthood to regulate separate functions: DAF-2/InsR, but not DAF-7/TGF-β, was thought to regulate longevity (Kenyon et al., 1993; Larsen et al., 1995). However, later, it was shown that matricide of DAF-7 pathway mutants obscured their extended lifespan phenotype (Shaw et al., 2007). The matricide resulted from an egg-laying defect where embryos would hatch internally. When lifespans were conducted with the addition of FUdR, a significant increase in lifespan was seen for *daf-7*, *daf-1*, *daf-4*, *daf-8* and *daf-14* mutants, compared to WT (Shaw et al., 2007). The increase in lifespan requires DAF-16/FOXO, a downstream transcription factor of the DAF-2/insulin signaling pathway. This indicates that DAF-7/TGF-β regulates longevity through DAF-2/insulin signaling (Shaw et al., 2007). An additional instance of DAF-2/insulin and DAF-7/TGF-β signaling crosstalk regulating lifespan is through HSF-1. The lifespan extension of *daf-7(e1372)* mutant animals was strongly, but partially, suppressed by the *hsf-1(sy441)* mutation, indicating that HSF-1 is regulated by DAF-7 (Barna et al., 2012). It is already known that HSF-1 is required for lifespan extension in IIS mutants (Hsu et al., 2003), indicating that HSF-1 may be connecting IIS and TGF-β pathways with respect to aging. Another regulator of aging that is downstream of DAF-7 is DAF-9 (Gerisch et al., 2001), which decreases lifespan by inhibiting DAF-12, a nuclear hormone receptor (Jia et al., 2002).

With respect to reproductive aging, there is little evidence that DAF-7 plays a role. However, it has been shown that inactivation of DAF-3 extends reproductive span (Wang et al., 2014).

## DAF-7 and the microbiome

DAF-7 signaling influences microbiome selection, though to a lesser degree than DBL-1. A large synthetic microbiome was created with 63 bacterial strains that reflect most of the core families found in wild *C. elegans* microbiomes. Over 38 wild *C. elegans* isolates were placed on plates with this synthetic microbiome at the first larval stage. After 120 h, the microbiome compositions could be quantified and categorized; three types emerged. A commensal member of the wild *C. elegans* microbiome is *Ochrobactrum*, which frequently became the dominant microbiome species in 28 of the 38 wild *C. elegans* strains assayed. Host insulin signaling is responsible for driving *Ochrobactrum* to establish microbiotic dominance (Zhang F. et al., 2021). The animals with an *Ochrobactrum*-dominant gut microbiome showed increased expression of DAF-8 and DAF-14 Smads that might be associated with upregulated DAF-7 signaling. Further study is needed to identify how DAF-7 contributes to this microbiome selection.

## DAF-7 and immunity

As described earlier, there are several mechanisms employed by organisms in host-pathogen interactions. DAF-7 has strongly been implicated in innate immunity with regards to pathogen avoidance. Odorant molecules or other metabolites secreted by pathogenic bacteria are one of the causes of aversive behavior in *C. elegans*. For example, *P. aeruginosa* produces the secondary metabolites phenazine-1-carboxamide and pyochelin. Chemosensory detection of these metabolites activates a G-protein-signaling pathway in ASJ neurons, which results in secretion of DAF-7. This activation of DAF-7/TGF-β signaling correlated with pathogen avoidance of *P. aeruginosa* (Meisel et al., 2014). A later paper showed that while chemosensation of these metabolites led to induction of DAF-7 in ASJ neurons, detection of these metabolites was not required for avoidance behavior (Singh and Aballay, 2019b). Instead, intestinal colonization by PA14, and subsequent intestinal bloating, was required for DAF-7-mediated pathogen avoidance (Singh and Aballay, 2019b; 2019a). Furthermore, *daf-7(e1372)* mutant animals show a partial loss of avoidance of PA14 (Singh and Aballay, 2019a) and loss of preference of *E. coli* over *P. aeruginosa* (Singh and Aballay, 2019b). Two other odorant molecules secreted by pathogenic bacteria are 2-nonanone and 1-undecene, which cause activation of the unfolded protein response of the endoplasmic reticulum (UPR^ER^) when detected (De-Souza et al., 2022). DAF-7 has been shown to be required for avoidance of 2-nonanone (Harris et al., 2019), and required for UPR^ER^ activation in response to 1-undecene (De-Souza et al., 2022). Interestingly, a 24 h exposure to 1-undecene in WT animals had a hormetic effect, causing extended lifespan. This effect was mediated through the DAF-7-dependent UPR^ER^ response (De-Souza et al., 2022) and indicates that induction of an immune response can extend lifespan.

DAF-7 expression in ASI neurons has been shown to be necessary for pathogen avoidance. AMPylase FIC-1 overexpression suppresses DAF-7 production in ASI neurons, which results in decreased pathogen avoidance (Hernandez-Lima et al., 2022). Another study found that exposure to a pathogen’s isolated and purified small RNAs was sufficient for *C. elegans* to exhibit avoidance behavior, as well as in four subsequent progeny generations (Kaletsky et al., 2020). Animals that experience a brief exposure and subsequent escape from PA14 transmit learned PA14 avoidance epigenetically to progeny and grandprogeny. This “training” encourages their survival. Transgenerationally inherited pathogen avoidance is mediated by Piwi/PRG-1 Argonaute and TGF-β signaling, specifically DAF-7 expression in the ASI neurons (Moore et al., 2019), demonstrating again the critical requirement for DAF-7 expression in the ASI neurons. These last two studies highlight the benefits of *C. elegans* as a model system to study innate immunity, where the short lifespan allows for transgenerational studies to be done with greater ease.

With regards to transcriptional response to pathogens, a 5 h exposure to *P. aeruginosa* PA14 is sufficient to upregulate DAF-7 mRNA levels in young adults (Singh and Aballay, 2019a). DAF-7 induction using a GFP reporter is seen in ASI neurons after 24 h of PA14 infection, or 24 h of PA14 small RNA exposure (Kaletsky et al., 2020). DAF-7::GFP induction is also seen in ASJ neurons (Meisel et al., 2014). However, one study found a decrease in DAF-7::GFP after a 4 h PA14 infection at the second larval stage (Jensen et al., 2010). This stage-specific difference presumably would increase dauer pheromone production, which would encourage dauer formation. It is possible this is an immune strategy, however further studies are necessary to elucidate this. Another instance of transcriptional response is how DAF-7 mediates a defense response triggered by detection of hydrogen peroxide. Across many species, hydrogen peroxide is secreted from cells, to attack or to defend against other species. *C. elegans* encounter hydrogen peroxide in the wild from plant matter, as well as from bacterial pathogens, which secrete the compound and can cause damage to the worms. Thus, *C. elegans* require robust resistance mechanisms against hydrogen peroxide to promote their own survival. This peroxide resistance is regulated by DAF-7 secretion from ASI neurons, received by interneurons, and followed by transcription of insulin genes. DAF-2 is then activated by the insulins, which independently inhibit DAF-16 and SKN-1, resulting in improved peroxide resistance (Schiffer et al., 2020). Interestingly, this DAF-7 and IIS cascade depends on the presence of *E. coli*. When *E. coli* is abundant, a freeloading strategy will be taken, where animals will not induce their own hydrogen peroxide degrading catalases, and instead rely on catalases produced by *E. coli*. In the absence of *E. coli*, a self-defense strategy will be taken, and catalases will be induced without reliance on gut microbiota (Schiffer et al., 2020). This affirms how important host-environment dynamics are in regulating immune response, and ultimately, survival.

## TIG-2, TIG-3, and UNC-129

**FIGURE 2:**
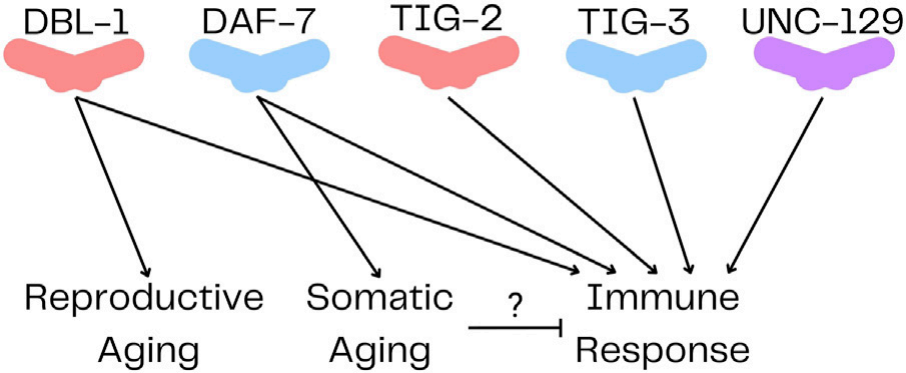
TGF-β Ligands in *Caenorhabditis elegans*. DBL-1 and TIG-2 are members of the BMP family; DAF-7 and TIG-3 are related to TGF-β/Activin; and UNC-129 is more divergent. All five ligands have been shown to regulate immune response, while only DBL-1 regulates reproductive aging, and DAF-7 regulates somatic aging.

Until very recently, there were no published functions for TIG-2 or TIG-3. However, TIG-2, TIG-3 and UNC-129 were recently shown to play a role in neuronal migration (Baltaci et al., 2022). In this function, they act non-redundantly through an atypical signaling pathway. The ligands act solely through SMA-6, the type I receptor, without a type II receptor, to regulate neural development (Baltaci et al., 2022). The pathways and functions of TIG-2, TIG-3 and UNC-129 are still being elucidated, and this recent work indicates that they may mediate signals differently from DAF-7 and DBL-1. Furthermore, a function for TIG-2 in regulating neuromuscular junctions (NMJs) was recently identified (Cheng et al., 2022). They found that *tig-2* mutants had slower locomotion, increased cholinergic synapse density, decreased NMJ neurotransmitter release, as well as decreased muscle mitochondria and ATP production. In addition to these developmental functions for TIG-2 and TIG-3, another recent study demonstrated that all five TGF-β ligands are involved in regulating the *C. elegans* innate immune response (Ciccarelli et al., 2023b) (Figure 2). Mutations in any of these ligands result in decreased survival on *P. luminescens*. Genetic evidence and structural modeling suggest that in this role, TIG-2 and TIG-3 work together while DBL-1 and DAF-7 work with each other (Ciccarelli et al., 2023b). This result shows unanticipated cooperativity between TGF-β/Activin and BMP family ligands and has implications for how an acute signaling response can be distinguished from developmental signaling.

## TGF-β signaling crosstalk with IIS

The canonical *C. elegans* IIS pathway begins with insulin-like ligands, which bind and activate or inhibit DAF-2. Receptor activation recruits phosphoinositide-3 kinase, AGE-1/PI3K, which initiates a signaling cascade that includes PIP_3_, and the serine/threonine kinases PDK-1, AKT-1, and AKT-2 (Dorman et al., 1995; Morris et al., 1996; Paradis and Ruvkun, 1998; Paradis et al., 1999). Then, transcription factors are inhibited, including DAF-16/FOXO and SKN-1/Nrf2, preventing activation of downstream target genes that promote longevity. Secondary transcriptional outputs of IIS include the heat shock transcription factor HSF-1, which is activated by the IIS cascade (Morley and Morimoto, 2004; Chiang et al., 2012) and promotes longevity by cooperating with DAF-16 (Hsu et al., 2003). DAF-2 also activates TOR signaling, which inhibits PHA-4/FoxA to restrict normal growth and longevity (Sheaffer et al., 2008). Separately, the transcription factor PHA-4 was identified as mediating longevity via dietary restriction (Panowski et al., 2007).

Mutations in *daf-2* extend lifespan by decreasing IIS activity and the PI3K cascade, thus activating the transcription factors and downstream targets. Decreased IIS results in changes to proteostasis, RNA homeostasis, oxidative stress, pathogen resistance, lipid and amino acid metabolism and endocrine signaling, all of which contribute to the longevity phenotype (Lee and Lee, 2022). The identification of this longevity pathway in *C. elegans* demonstrated the utility of *C. elegans* for discovery in the field of aging, and IIS-dependent aging has been shown to be conserved across many species, including rodent models and humans (Kenyon, 2010).

DBL-1 has numerous modes of interaction with the longevity-regulating IIS pathway. Both DBL-1 and DAF-2/InsR regulate reproductive aging, modulating the length of time before female reproductive capacity ceases (Luo et al., 2009; 2010). DBl-1 and DAF-2/InsR also regulate body size (Qi et al., 2017; Clark et al., 2018). In both roles, the pathways act independently. In the regulation of lipid metabolism, DBL-1 signaling negatively regulates DAF-2/InsR signaling, so that loss of *daf-2* is genetically epistatic to loss of *dbl-1*, or its signaling components (Clark et al., 2018; 2021). The DBL-1 pathway transcriptionally regulates several insulin-like ligand genes (Liang et al., 2007; Luo et al., 2010). The DBL-1 pathway also regulates a subset of DAF-16/FOXO transcriptional targets, like *fat-6* and *fat-7* (Liang et al., 2007). SMA-3 acts upstream of insulin-like ligand INS-4 in the regulation of fat storage, which carries out homeostatic functions through DAF-16/FOXO (Clark et al., 2021). An antagonistic interaction between DBL-1 signaling and DAF-2/InsR was revealed by the partial suppression of *daf-2* phenotypes of longevity, dauer formation, and autophagy by loss of *dbl-1* or its signaling components (Clark et al., 2018). These two pathways have also been shown to interact in first larval stage (L1) arrest and Q cell divisions (Kaplan et al., 2015; Zheng et al., 2018).

The DAF-7 pathway also has many instances of crosstalk with IIS. DAF-7 signaling acts upstream of DAF-2/InsR signaling (Shaw et al., 2007), and interacts in a cooperative fashion with DAF-2/InsR in dauer formation, lipid metabolism, and longevity (Ogg et al., 1997; Narasimhan et al., 2011). It is hypothesized that these two pathways cooperate so that multiple signal inputs can be used to inform dauer and lifespan decisions (Shaw et al., 2007). Some of this signal integration may occur through DAF-12/nuclear hormone receptor (Antebi et al., 2000; Snow and Larsen, 2000). The DAF-7 pathway functions epistatically with IIS in regulating nictation behavior (Lee et al., 2017). Nictation is a waving behavior that enables dauer animals to “hitchhike” on more mobile animals as a way to move to an area with more favorable conditions, such as lower population density and an increased abundance of food. Furthermore, expression of several insulin-like ligands, such as INS-7 and INS-18, are regulated by the DAF-7 pathway (Liu et al., 2004; Shaw et al., 2007; Narasimhan et al., 2011). Lastly, the DAF-7 pathway promotes secretion of insulin-like ligand DAF-28 (Park et al., 2012).

## Discussion

Studies using *C. elegans* have greatly contributed to understanding the functions and mechanisms of TGF-β signaling. This review focuses on the multifaceted connections between TGF-β/Activin and BMP signaling and their roles in regulating longevity and immunity in *C. elegans*. Interestingly, delving into these physiological aspects has unveiled many instances of non-canonical signaling, which may have implications for mammalian signaling and thus deserve more extensive study. Because TGF-β signaling is one of the key pathways co-regulating aging and immunity (Fabian et al., 2021), mechanisms identified in *C. elegans* are anticipated to shed light on these processes in all organisms. Notably, one study examined protein signatures of centenarians and identified TGF-β signaling, as well as IIS, as hallmark pathways of healthy aging (Sebastiani et al., 2021). Leveraging the advantages of the *C. elegans* system, research can now explore additional associations between aging and immunity, the relationship of the microbiome with these physiologies, crosstalk between TGF-β and IIS pathways, and mechanisms that differentiate TGF-β/Activin and BMP family activities. Additionally, the short-lived nature of *C. elegans* offers a unique opportunity to investigate transgenerational effects, paving the way for further groundbreaking studies in this field.
